# Supplementary material for: Structural and functional characterization of a putative de novo gene in Drosophila
Source: Nat Commun. 2021 Mar 12;12:1667. doi: 10.1038/s41467-021-21667-6 (PMC7954818; doi:10.1038/s41467-021-21667-6)
Supplement: Supplementary file 1 — Supplementary Information [file 41467_2021_21667_MOESM1_ESM.pdf]

Supplementary Information for "Structural and functional characterization of a putative *de novo* gene in *Drosophila*"

Andreas Lange[1+], Prajal H. Patel[2+], Brennen Heames[1], Adam M. Damry[3], Thorsten Saenger[4], Colin J. Jackson[3], Geoffrey D. Findlay[2*], Erich Bornberg-Bauer[1*]

[1]Institute for Evolution and Biodiversity, University of Muenster, Germany
[2]Department of Biology, College of the Holy Cross, Worcester, MA, USA
[3]Research School of Chemistry, ANU College of Science, Canberra, Australia
[4]Department of Pediatric Kidney, Liver and Metabolic Diseases, Hannover Medical School, Hannover, Germany

+ these authors contributed equally
[*] corresponding authors: ebb.admin@uni-muenster.de, gfindlay@holycross.edu

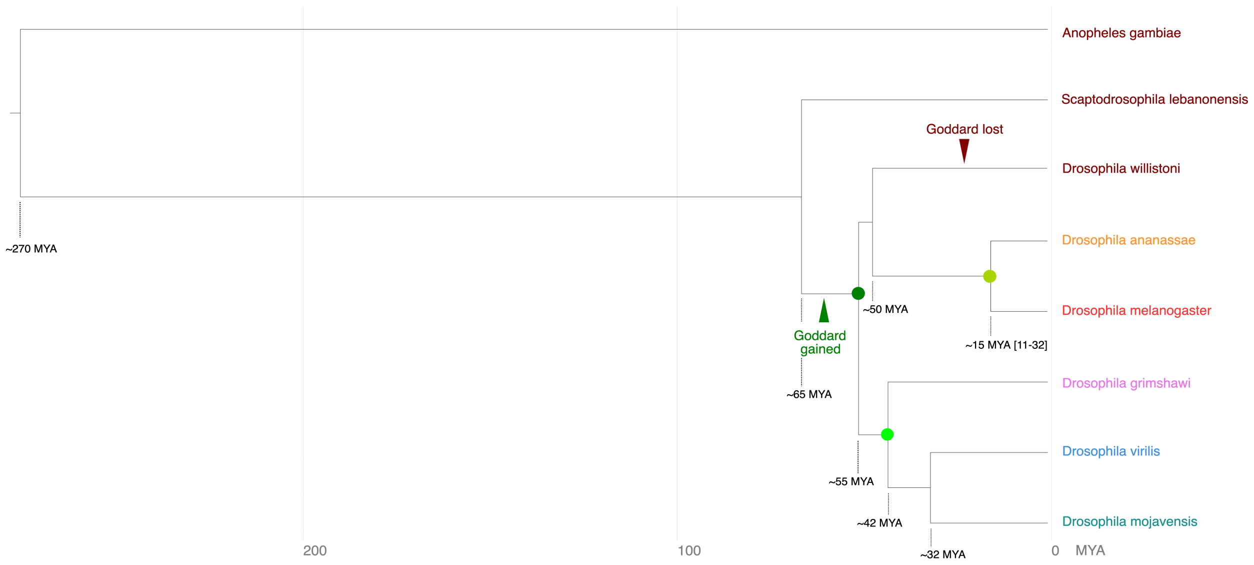

Supplementary Figure 1: Phylogenetic tree showing the origination of the *gdrd* gene at the base of *Drosophila*. Gdrd appears to have emerged within an intron of the highly conserved omega gene, 50 million years ago. We previously ruled out the presence of the *gdrd* coding sequence in outgroup genomic regions syntenic to this intron [1]. Similarly, applying the same methodology, we find no evidence for an established *gdrd* gene in the recently released *Scaptodrosophila lebanonensis* genome. This suggests that *gdrd* is no older than 65 My. Reconstructed ancestral nodes are marked with green circles (colour matching main text Figure 6). Divergence times are best estimates from Obbard et al. (2012) and Russo et al. (2013) [2, 3].

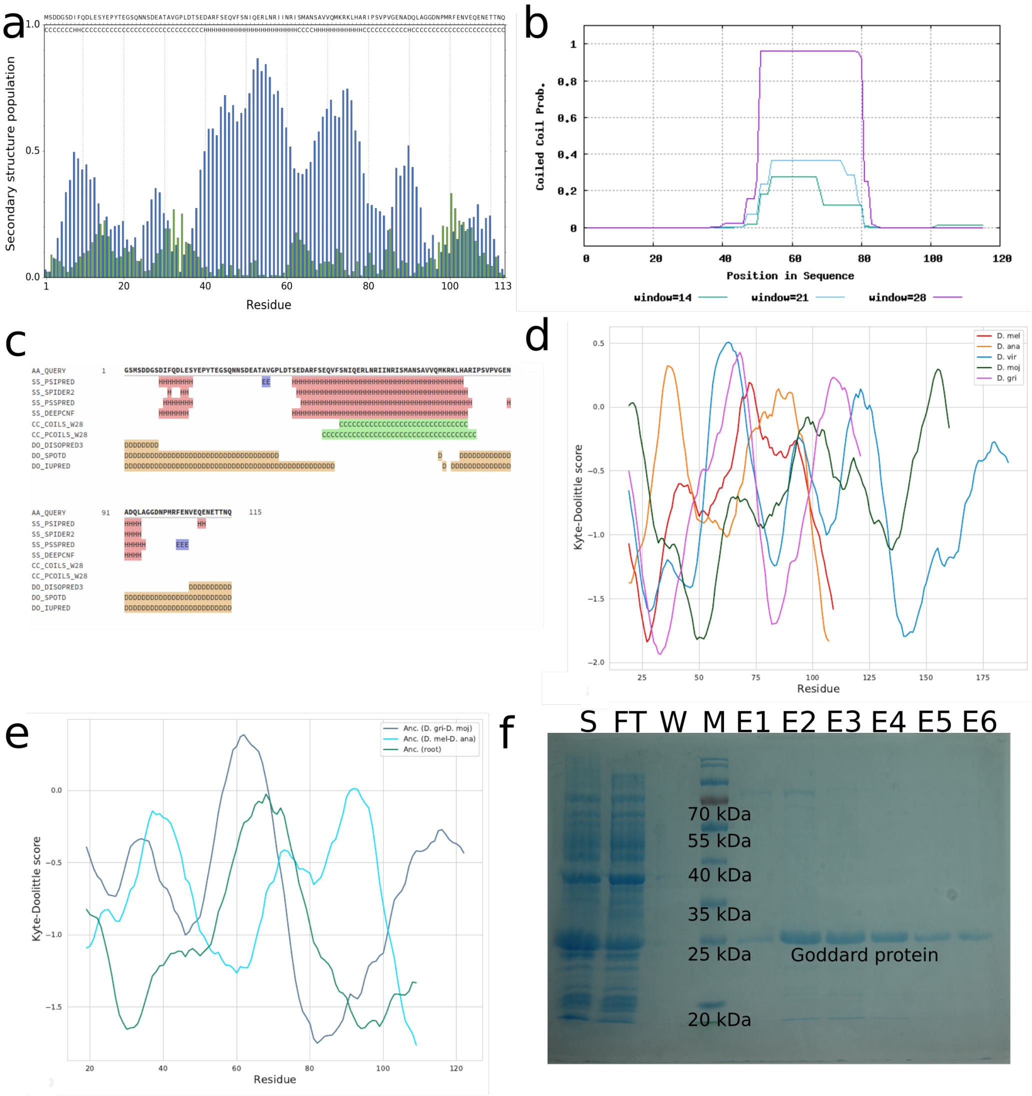

Supplementary Figure 2: Structural predictions for Gdrd a) s2D[4], b) PCOILS[5], and c) Quick2D[6]. **Kyte-Doolittle plots illustrating the hydrophobicity** of d) Gdrd orthologs in all five *Drosophila* species and e) reconstructed sequences of Gdrd from three ancestral nodes. Regions of positive hydrophobicity are mainly isolated to the core of the protein in all sequences and correspond to α helix predictions. f) **SDS-Gel of Gdrd purification**. Samples are (S) cell-free extract (before adding Ni^{2+} beads); (FT) Flow through, (W) wash, (E1 to E6) elution steps with buffer B. Size of 6xHis-Gdrd is predicted to be 15 kDa, however, always runs at 25 kDa in TGS-SDS-gels or Bis-Tris gels. For mass detection (i) a band from elution fractions (for example like E2) was cut from SDS-Gel and analyzed via trypsin MALDI-TOF (Prof. König, Core Unit Proteomics, UKM Muenster) and (ii) a sample of combined elution fractions was taken for ESI-MS (Susan Hawat, Department of Plant Biochemistry and Biotechnology, WWU Muenster). Both measurements detected Gdrd protein (see mass spectrometry data, Zenodo DOI: 10.5281/zenodo.4476357 or Supplementary Data 1 and 2. SDS-PAGE of the purification of Gdrd was repeated three times with similar results. Source data are provided as a Source Data file.

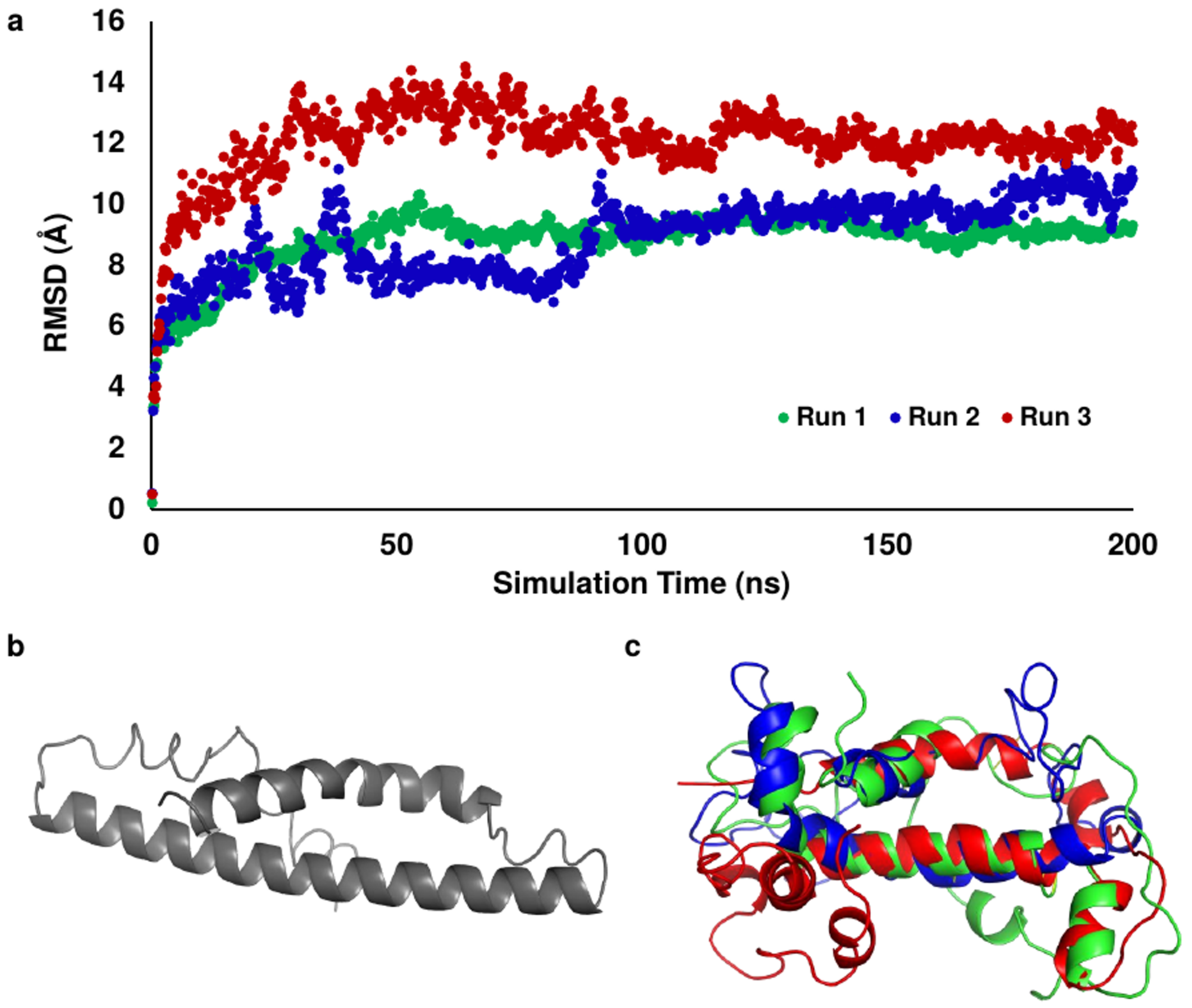

Supplementary Figure 3: MD simulation diversity. a) Plot of simulation RMSD versus time shows a rapid divergence from the simulation input structure [7, 8, 9]. Following distortion of the starting loops and helices, all three trajectories reach relatively stable RMSD values within the first 100 ns of the simulation. b) Input structure obtained from the QUARK webserver[10]. c) Overlay of the endpoint structures from the three simulation replicates demonstrates the high variability observed in the protein C-terminus and loops. Positioning of the N-terminal helix and central helix however remains consistent.

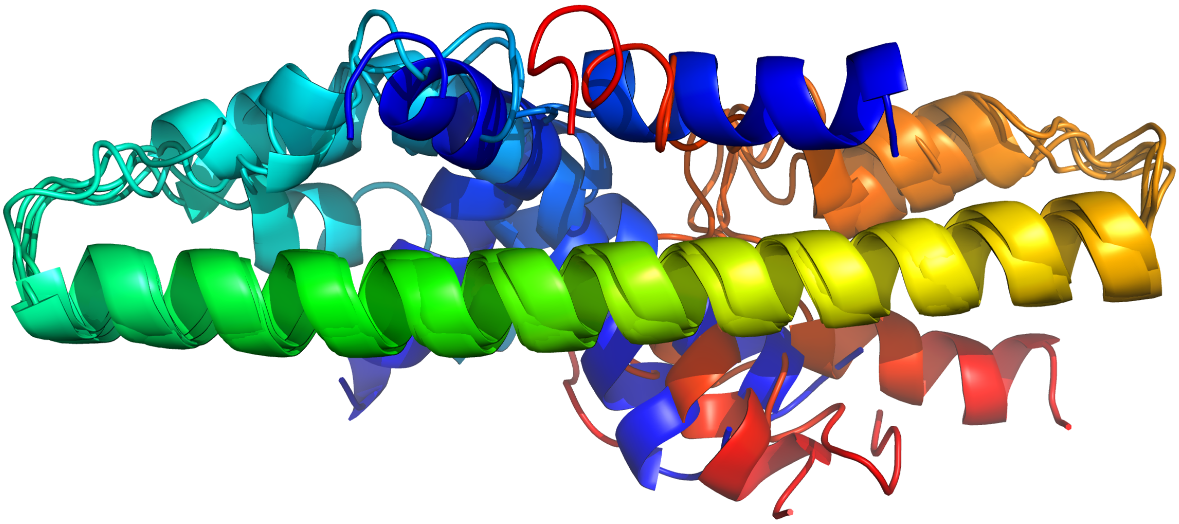

Supplementary Figure 4: Alignment of top 5 QUARK predicted structures of Gdrd.[10] As expected, there are some differences at the termini of Gdrd, but the prediction of the core α-helix is highly consistent (pairwise RMSD of 2.5 to 3.0 Å between residues 37-77). Figure made using PyMOL [11].

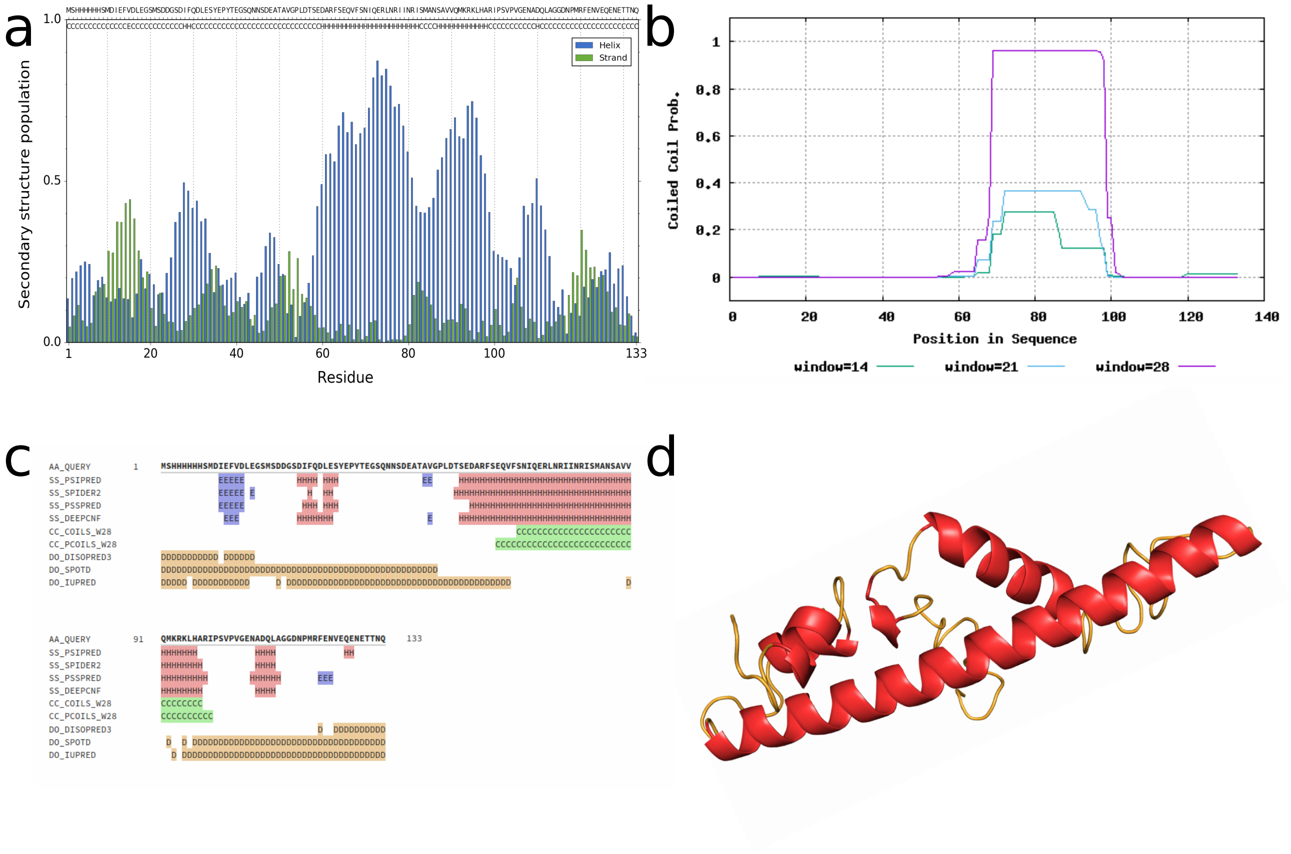

Supplementary Figure 5: Structural predictions for the 6xHis-tagged *Gdrd* sequence using a) s2D [4], b) PCOILS [5], c) Quick2D [6], and d) QUARK (helix shown in red) [10]. Predictions are highly similar to the untagged version of Gdrd (Figure S1). The β-sheet region predicted by Quick2D (residues 15-18) is part of the 6x-His-tag.

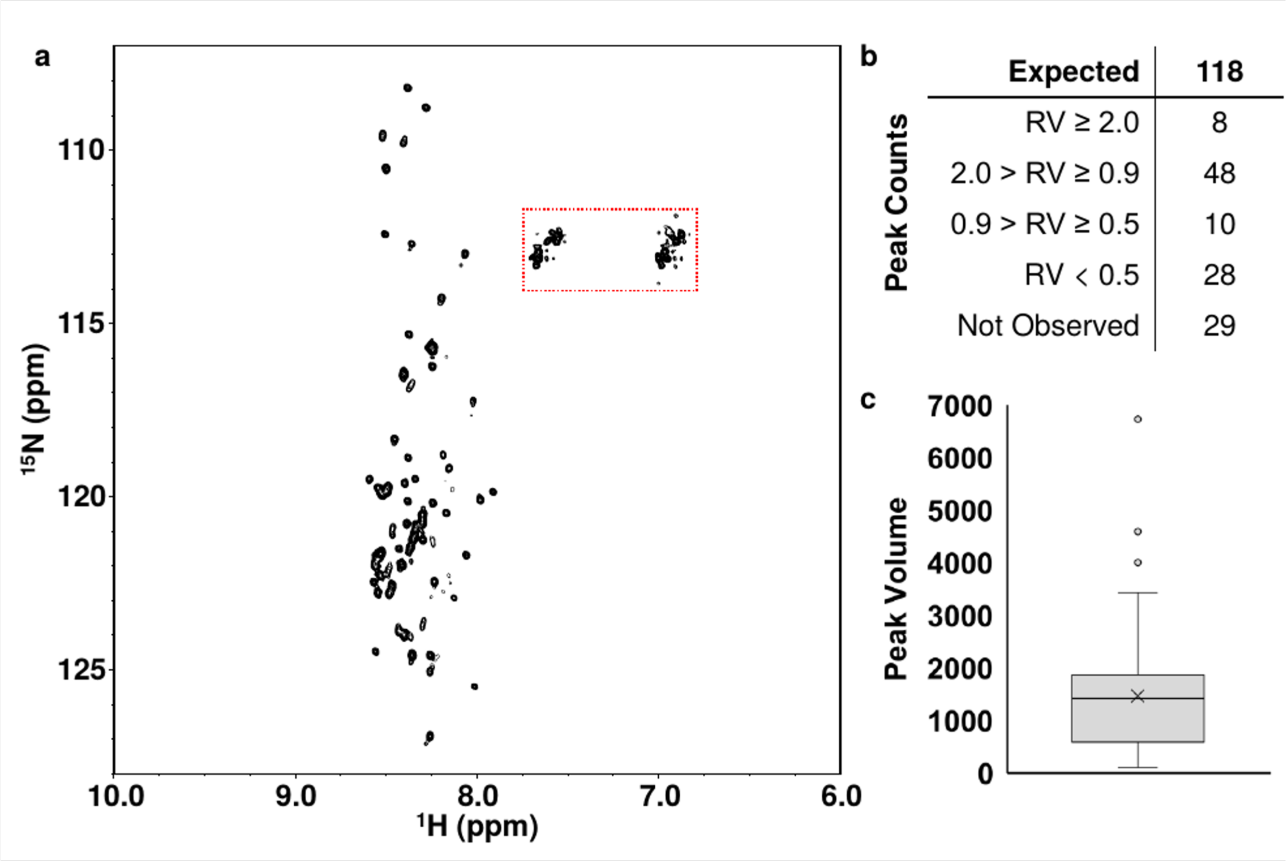

Supplementary Figure 6: NMR analysis of Gdrd suggests a partially-ordered helical structure. a) ^{1}H-^{15}N heteronuclear multiple quantum coherence (HMQC) spectrum of Goddard. The relatively poorly dispersed set of high-intensity peaks suggests that the ordered segments of the protein adopt a low-diversity secondary structure, which supports the modeled structure and MD simulations that suggest that these ordered regions are dominantly helical. Several additional peaks that are strongly broadened are also observed, suggesting that the remaining structure is highly flexible. Glutamine and asparagine side-chain peaks, boxed in red, were not included in further analyses. b) Count of peaks as a function of median-normalized relative volume (RV) demonstrates that roughly 40-50$\%$ of Goddard adopts an ordered structure (defined by the interval $2.0 \geq \text{RV} \geq 0.5$). c) Box plot of the peak volume distribution for observable peaks highlights the population of broadened, low-volume peaks that are indicative of conformational flexibility in the protein structure. Minimum (Lower whisker bound): 98.602, Quartile 1 (Lower box bound): 578.9263, Median: 1414.34, Mean: 1463.202, Quartile 3 (Upper box bound): 1882.665, Upper whisper bound: 3435.566, Maximum (inc. outliers): 6731.656. The three points defined as outliers were >1.5 times the interquartile range above the third quartile. Source data are provided as a Source Data file.

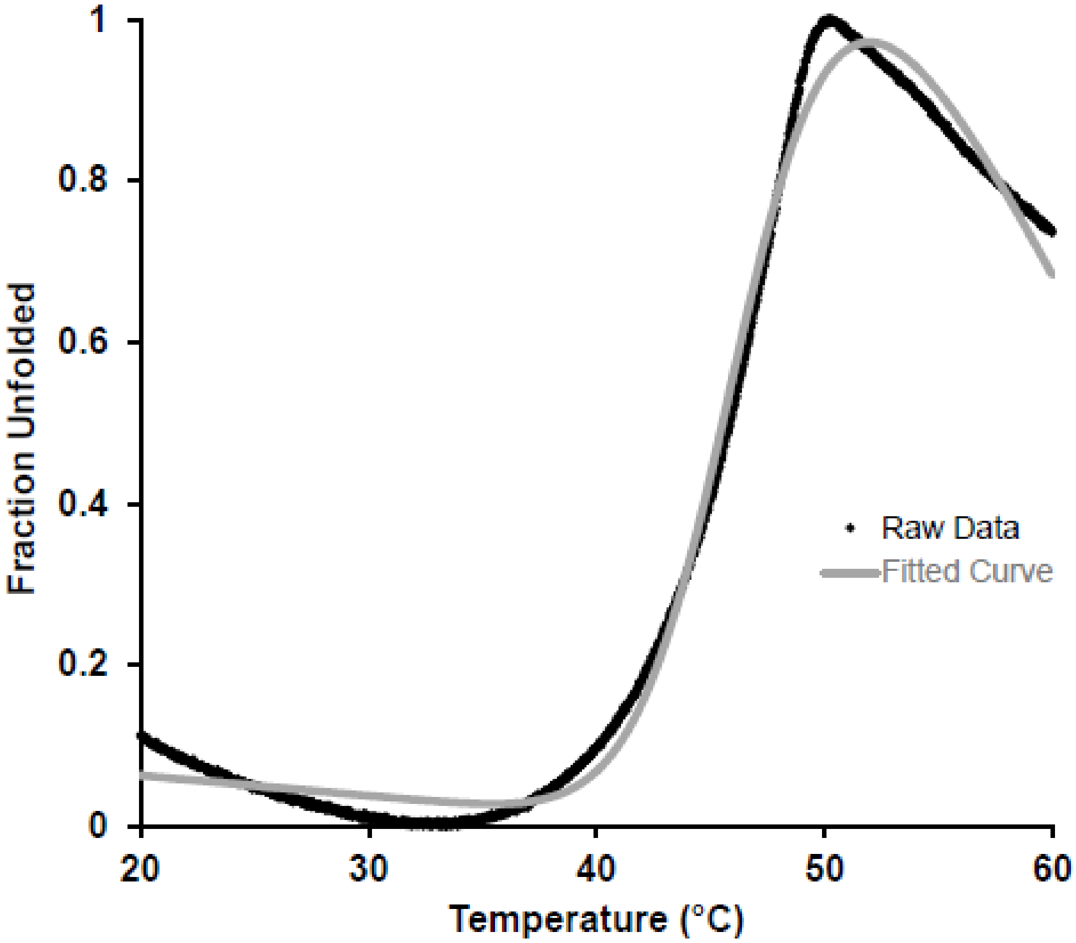

Supplementary Figure 7: Thermal unfolding assay of Gdrd. Gdrd thermal unfolding data was obtained using a SYPRO orange based unfolding assay (see Methods). Raw fluorescence data was converted to a fraction unfolded value under the assumption that the lowest fluorescence signal obtained corresponds to a fully folded protein and the highest fluorescence signal to a fully unfolded protein with all dye binding sites exposed. A linear slope corrected sigmoid was fit to the data and used to determine the melting temperature, 47.3°C +/- 0.9°C (average of 12 replicates +/- 1 standard deviation). Source data are provided as a Source Data file.

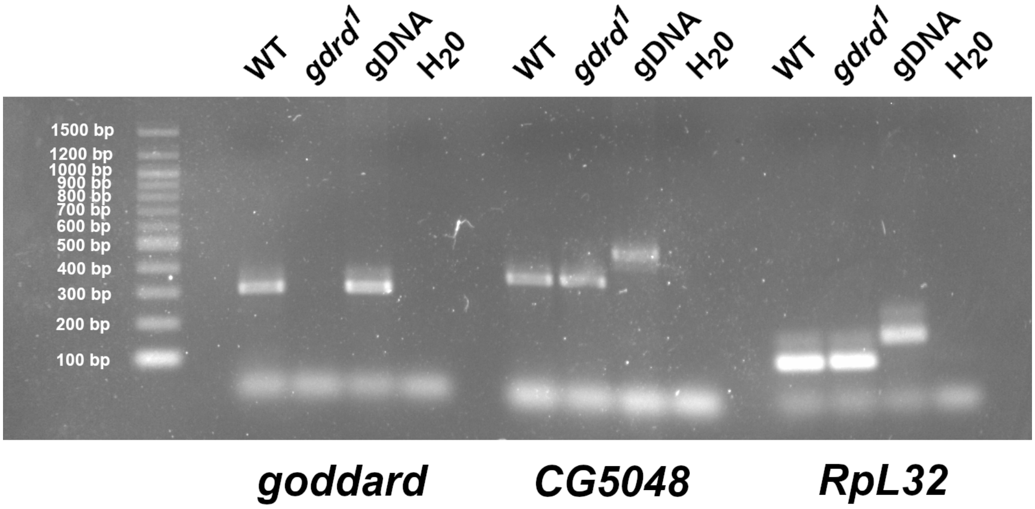

Supplementary Figure 8: *gdrd^1* mutation specifically affects *gdrd* expression. (a) We performed RT-PCR analysis of *gdrd*, *CG5048*, and *RpL32* mRNA levels in wild type (*w^{1118}*) and *gdrd^1 flies*. *gdrd* expression is undetectable in *gdrd^1* flies, while the expression of adjacent gene, *CG5048*, is unaffected in the *gdrd^1* deletion mutant. Amplification using genomic DNA (gDNA) and water were performed as positive and negative controls respectively. Data set represents a single replicate. Source data are provided as a Source Data file.

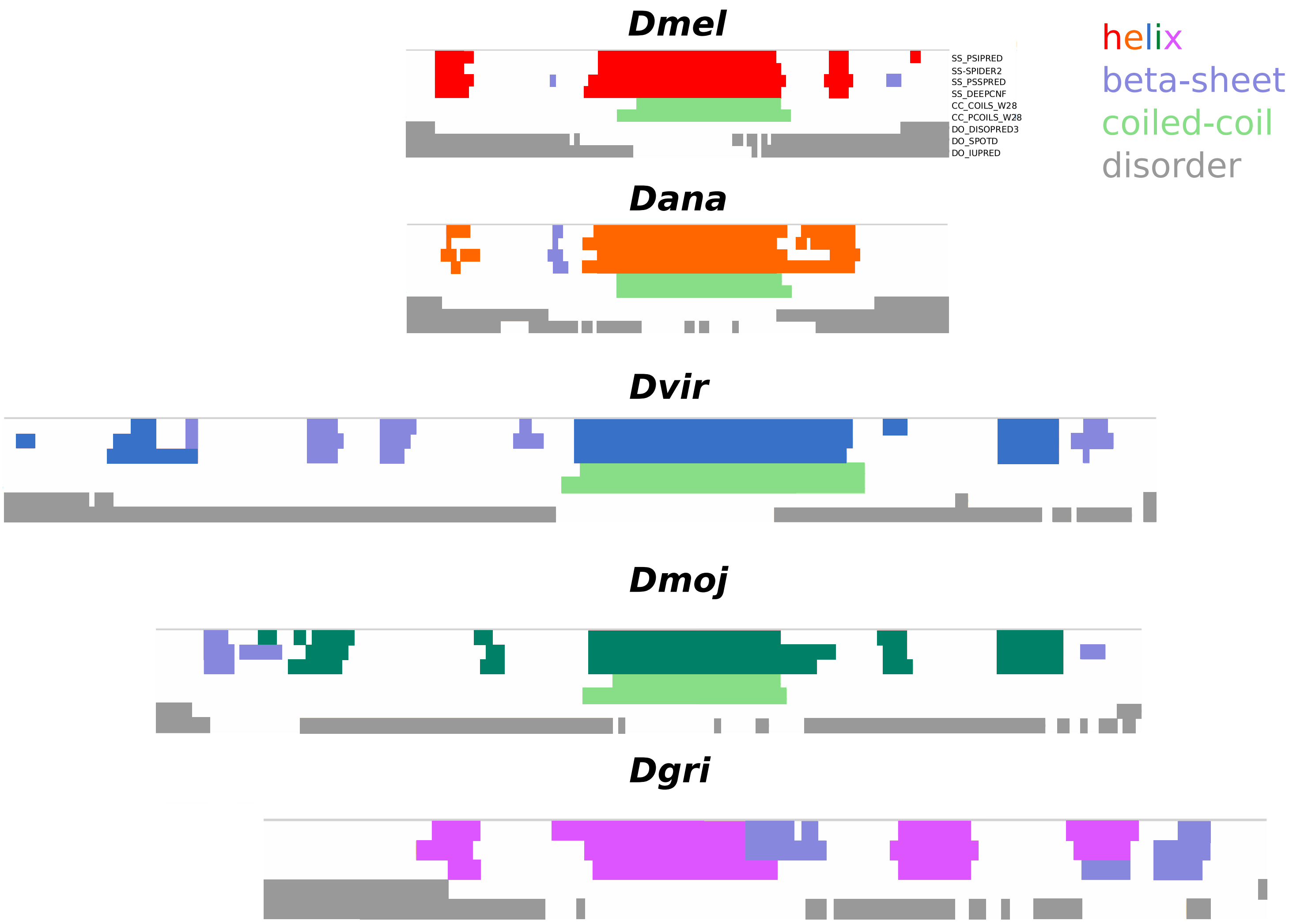

Supplementary Figure 9: Quick2D predictions of Gdrd orthologs from *Dmel*, *Dana*, *Dvir*, *Dmoj*, and *Dgr*. Each block of color represents the linear amino acid sequence of Gdrd from different species. Helices are shown with a different colour in each species, β-sheets in lilac, disordered regions in grey [6].

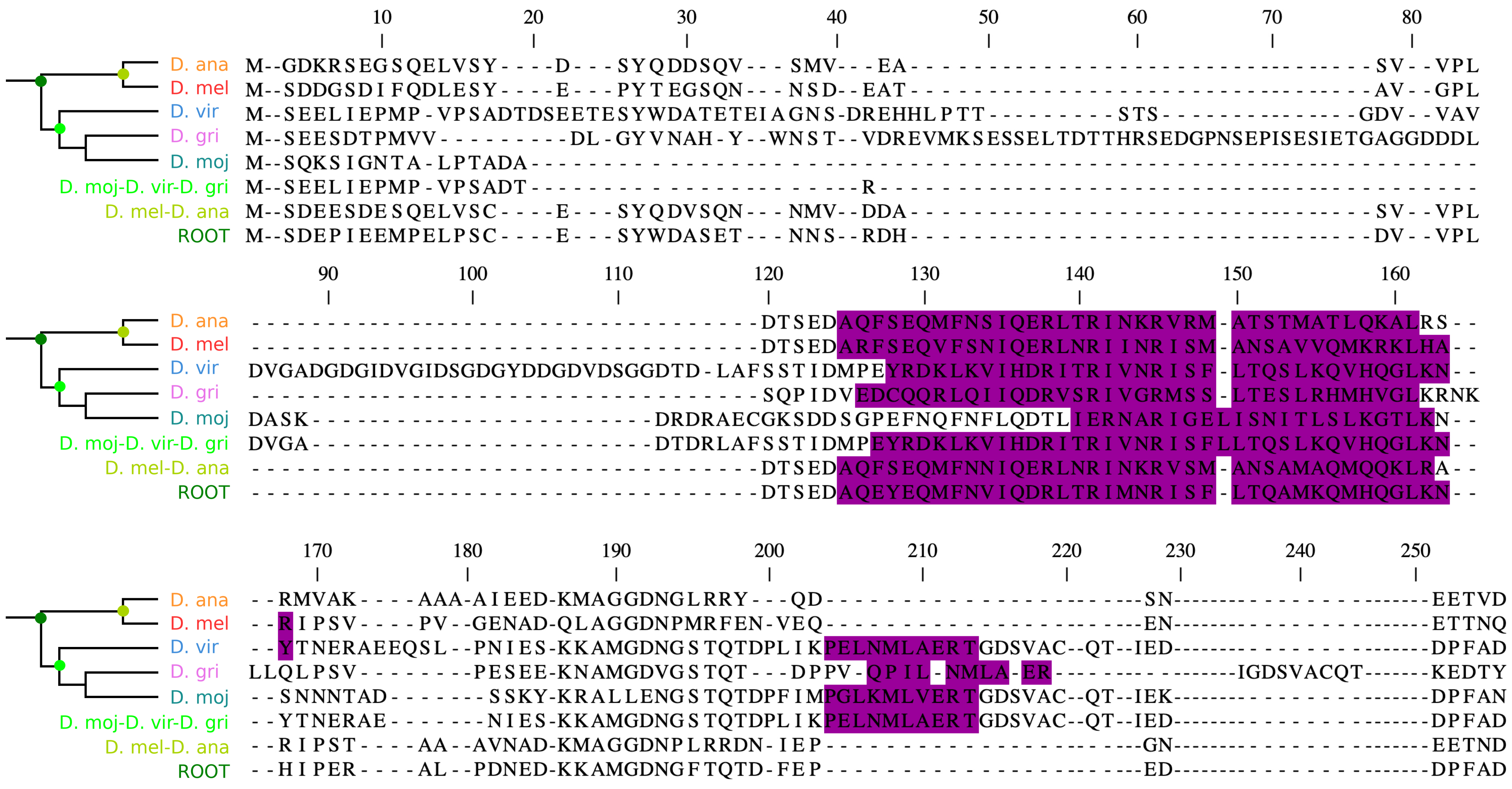

Supplementary Figure 10: Sequence alignment of Gdrd orthologs from five *Drosophila* species and three reconstructed ancestral sequences. Secondary structure prediction using s2D[4] indicates highly conserved α-helical character in extant and ancestral sequences (highlighted in purple).

Primer name	Primer Sequence 5'– 3'	experimental use
Gdrd RT F	TCCAAGACCTTGAAAGCTACG	RT PCR
Gdrd RT R	TGGTTGGTGGTTTCGTTTTC	RT PCR
CG5048 RT F	GGAGTCAATGGTTGTCAATA	RT PCR
CG5048 RT R	ACATTTTGTCAGCAGTCATT	RT PCR
RPL32 RT F	CACCAGTCGGATCGATATGC	RT PCR
RPL32 RT R	CGATCCGTAACCGATGTTG	RT PCR
Gdrd Rescue F1	GGCATGTCGACCTCGAGTACC CGGGAGCTCGAATTCTAGATA AGGAAAAGCGAAGCACAC	Tagged gdrd rescue (for amplification of upstream regulatory sequences and CDS)
Gdrd Rescue R1	ATGGGTAAAAGATGCGGCCTC CACCGCGGTGGAGATCCATTT GGTTGGTGGTTTCGTTTT	Tagged gdrd rescue (for amplification of upstream regulatory sequences and CDS)
Gdrd Rescue F3	TGATATAAGAACATTTTTATA TTTCTCATTTTCAAAAATGTATA AATTTATTGTATTTAT	Tagged gdrd rescue (for amplification of downstream regulatory sequences)
Gdrd Rescue R3	ATTGCCGGCGATATCGGATCC ACCGGTGCCTAGGCGCGCCTG CAGAAGATGATTTAGAAA	Tagged gdrd rescue (for amplification of downstream regulatory sequences)
Gdrd Rescue F2	ATGGATCTCCACCGCGGTGGA GGCCGCA	Tagged gdrd rescue (for amplification of HA tag)
Gdrd Rescue R2	CATTTTTGAAAATGAGAAATAT AAAAATGTTCTTATATCACGTG GACCGGTGTCCGCCAT	Tagged gdrd rescue (for amplification of HA tag)
Gdrd F	GCGCGCGGATCCATGTCGGAC GACGGATCTGATATAT	For bacterial expression/purification of Gdrd (BamH1 site)
Gdrd R	CGCGCGAAGCTTTTATTGGTTG GTGGTTTCGTTTTCTT	For bacterial expression/purification of Gdrd (HindIII site)

Supplementary Table 1: Oligonucleotides used for molecular cloning and RT PCR.

Species	accession code	hyperlink
Drosophila melanogaster	NP_648713.1	https://www.ncbi.nlm.nih.gov/protein/NP_648713.1
Drosophila erecta	XP_001972744.1	https://www.ncbi.nlm.nih.gov/protein/XP_001972744.1
Drosophila yakuba	XP_002094802.2	https://www.ncbi.nlm.nih.gov/protein/XP_002094802.2
Drosophila simulans	XP_002030500.1	https://www.ncbi.nlm.nih.gov/protein/XP_002030500.1
Drosophila rhopaloa	XP_016990426.1	https://www.ncbi.nlm.nih.gov/protein/XP_016990426.1
Drosophila eugracilis	XP_017068231.1	https://www.ncbi.nlm.nih.gov/protein/XP_017068231.1
Drosophila biarmipes	XP_016965957.1	https://www.ncbi.nlm.nih.gov/protein/XP_016965957.1
Drosophila takahashii	XP_016994458.1	https://www.ncbi.nlm.nih.gov/protein/XP_016994458.1
Drosophila suzukii	XP_016934355.1	https://www.ncbi.nlm.nih.gov/protein/XP_016934355.1
Drosophila elegans	XP_017122925.1	https://www.ncbi.nlm.nih.gov/protein/XP_017122925.1
Drosophila ficusphila	XP_017040312.1	https://www.ncbi.nlm.nih.gov/protein/XP_017040312.1
Drosophila kikkawai	XP_017019509.1	https://www.ncbi.nlm.nih.gov/protein/XP_017019509.1
Drosophila pseudoobscura	XP_002134768.1	https://www.ncbi.nlm.nih.gov/protein/XP_002134768.1
Drosophila persimilis	XP_002022284.1	https://www.ncbi.nlm.nih.gov/protein/XP_002022284.1
Drosophila miranda	XP_017135053.1	https://www.ncbi.nlm.nih.gov/protein/XP_017135053.1
Drosophila ananassae	XP_001956400.1	https://www.ncbi.nlm.nih.gov/protein/XP_001956400.1
Drosophila serrata	XP_020798253.1	https://www.ncbi.nlm.nih.gov/protein/XP_020798253.1
Drosophila bipectinata	XP_017109486.1	https://www.ncbi.nlm.nih.gov/protein/XP_017109486.1
Drosophila guanche	SPP76062.1	https://www.ncbi.nlm.nih.gov/protein/SPP76062.1

Supplementary Table 2: List of all accession codes used in this study.

References

[1] A. M. Gubala, J. F. Schmitz, M. J. Kearns, T. T. Vinh, E. Bornberg-Bauer, M. F. Wolfner, and G. D. Findlay, “The goddard and saturn genes are essential for Drosophila male fertility and may have arisen de novo,” *Molecular Biology and Evolution*, Jan. 2017.

[2] D. J. Obbard, J. Maclennan, K.-W. Kim, A. Rambaut, P. M. O’Grady, and F. M. Jiggins, “Estimating divergence dates and substitution rates in the drosophila phylogeny,” *Molecular Biology and Evolution*, vol. 29, no. 11, pp. 3459–3473, Nov. 2012.

[3] C. A. M. Russo, B. Mello, A. Frazão, and C. M. Voloch, “Phylogenetic analysis and a time tree for a large drosophilid data set (diptera: Drosophilidae),” *Zoological Journal of the Linnean Society*, vol. 169, no. 4, pp. 765–775, 2013.

[4] P. Sormanni, C. Camilloni, P. Fariselli, and M. Vendruscolo, “The s2d Method: Simultaneous Sequence-Based Prediction of the Statistical Populations of Ordered and Disordered Regions in Proteins,” *Journal of Molecular Biology*, vol. 427, no. 4, pp. 982–996, Feb. 2015.

[5] M. Gruber, J. Söding, and A. N. Lupas, “Comparative analysis of coiled-coil prediction methods,” *Journal of Structural Biology*, Fibrous Protein Structure, vol. 155, no. 2, pp. 140–145, Aug. 2006.

[6] L. Zimmermann, A. Stephens, S.-Z. Nam, D. Rau, J. Kübler, M. Lozajic, F. Gabler, J. Söding, A. N. Lupas, and V. Alva, “A Completely Reimplemented MPI Bioinformatics Toolkit with a New HHpred Server at its Core,” *Journal of Molecular Biology*, Computation Resources for Molecular Biology, vol. 430, no. 15, pp. 2237–2243, Jul. 2018.

[7] H. J. C. Berendsen, D. van der Spoel, and R. van Drunen, “GROMACS: A message-passing parallel molecular dynamics implementation,” *Computer Physics Communications*, vol. 91, no. 1, pp. 43–56, Sep. 2, 1995.

[8] S. Pronk, S. Páll, R. Schulz, P. Larsson, P. Bjelkmar, R. Apostolov, M. R. Shirts, J. C. Smith, P. M. Kasson, D. van der Spoel, B. Hess, and E. Lindahl, “GROMACS 4.5: A high-throughput and highly parallel open source molecular simulation toolkit,” *Bioinformatics*, vol. 29, no. 7, pp. 845–854, Apr. 1, 2013.

[9] M. J. Abraham, T. Murtola, R. Schulz, S. Páll, J. C. Smith, B. Hess, and E. Lindahl, “GROMACS: High performance molecular simulations through multi-level parallelism from laptops to supercomputers,” *SoftwareX*, vol. 1-2, pp. 19–25, Sep. 1, 2015.

[10] D. Xu and Y. Zhang, “Toward optimal fragment generations for ab initio protein structure assembly,” *Proteins*, vol. 81, no. 2, pp. 229–239, Feb. 2013.

[11] *The PyMOL molecular graphics system, version 1.2r3pre, schrödinger, LLC.*
