# Supplementary material for: Structural and functional characterization of a putative de novo gene in Drosophila
Source: Nat Commun. 2021 Mar 12;12:1667. doi: 10.1038/s41467-021-21667-6 (PMC7954818; doi:10.1038/s41467-021-21667-6)
Supplement: Supplementary file 2 — Description of Additional Supplementary Files [file 41467_2021_21667_MOESM2_ESM.pdf]

Description of Additional Supplementary Files

Supplementary Data 1: Mass data of Goddard protein. A single band from elution fraction E2 was cut from SDS-Gel and analyzed via trypsin MALDI-TOF (Prof. König, Core Unit Proteomics, UKM Muenster)

Supplementary Data 2: Mass data of Goddard protein. A sample of combined elution fractions of purifed Gdrd protein was measured via ESI-MS (Susan Hawat, Department of Plant Biochemistryand Biotechnology, WWU Muenster).
