# Supplementary material for: Structural and functional characterization of a putative de novo gene in Drosophila
Source: Nat Commun. 2021 Mar 12;12:1667. doi: 10.1038/s41467-021-21667-6 (PMC7954818; doi:10.1038/s41467-021-21667-6)
Supplement: Supplementary file 5 — Reporting Summary [file 41467_2021_21667_MOESM5_ESM.pdf]

nature research

Corresponding author(s): Geoffrey Findlay, Erich Bornberg-Bauer

Last updated by author(s): Feb 3, 2021

Reporting Summary

Nature Research wishes to improve the reproducibility of the work that we publish. This form provides structure for consistency and transparency in reporting. For further information on Nature Research policies, see our Editorial Policies and the Editorial Policy Checklist.

Statistics

For all statistical analyses, confirm that the following items are present in the figure legend, table legend, main text, or Methods section.

n/a	Confirmed	
☐	☒	The exact sample size (*n*) for each experimental group/condition, given as a discrete number and unit of measurement
☐	☒	A statement on whether measurements were taken from distinct samples or whether the same sample was measured repeatedly
☐	☒	The statistical test(s) used AND whether they are one- or two-sided<br>*Only common tests should be described solely by name; describe more complex techniques in the Methods section.*
☒	☐	A description of all covariates tested
☐	☒	A description of any assumptions or corrections, such as tests of normality and adjustment for multiple comparisons
☐	☒	A full description of the statistical parameters including central tendency (e.g. means) or other basic estimates (e.g. regression coefficient) AND variation (e.g. standard deviation) or associated estimates of uncertainty (e.g. confidence intervals)
☐	☒	For null hypothesis testing, the test statistic (e.g. F, t, r) with confidence intervals, effect sizes, degrees of freedom and P value noted<br>*Give P values as exact values whenever suitable.*
☒	☐	For Bayesian analysis, information on the choice of priors and Markov chain Monte Carlo settings
☒	☐	For hierarchical and complex designs, identification of the appropriate level for tests and full reporting of outcomes
☒	☐	Estimates of effect sizes (e.g. Cohen's *d*, Pearson's *r*), indicating how they were calculated

Our web collection on statistics for biologists contains articles on many of the points above.

Software and code

Policy information about availability of computer code

Data collection	No software has been used for data collection
Data analysis	Structural predictions: s2D (s2Dv2, http://www-cohsoftware.ch.cam.ac.uk/index.php/s2D, PyMOL v1.8.4.0, SALIGN vr202 (https://modbase.compbio.ucsf.edu/salign/), PSIPRED&PCOILS (https://toolkit.tuebingen.mpg.de/tools/pcoils), Kyte-Doolittle Expasy (https://web.expasy.org/protscale/), Quick2D, (https://toolkit.tuebingen.mpg.de/tools/quick2d), Quark (https://zhanglab.ccmb.med.umich.edu/QUARK2), TMalign (https://yanglab.nankai.edu.cn/mTM-align/), 3D-Blast (http://3d-blast.life.nctu.edu.tw/), Tango v2.3.1, PLAAC (http://plaac.wi.mit.edu); MD: Gromacs 2018.1, VMD 1.9.3; ASR: T-COFFEE v8.97, RAxML v8.2.12; NMR: NMRViewJ (OneMoonScientific), TopSpin v3.5 (Bruker); Fly data: Microsoft excel for Mac v16.16.27 (201012), ImageJ: Fiji v1.0, Kaleidagraph v4.1.3;<br>All generated data, codes, fasta files used are deposited here: Zenodo, https://doi.org/10.5281/zenodo.4476357

For manuscripts utilizing custom algorithms or software that are central to the research but not yet described in published literature, software must be made available to editors and reviewers. We strongly encourage code deposition in a community repository (e.g. GitHub). See the Nature Research guidelines for submitting code & software for further information.

Data

Policy information about availability of data

All manuscripts must include a data availability statement. This statement should provide the following information, where applicable:

- Accession codes, unique identifiers, or web links for publicly available datasets
- A list of figures that have associated raw data
- A description of any restrictions on data availability

Accession codes for publicly available datasets have been deposited online together with the ASR data on Zenodo: https://doi.org/10.5281/zenodo.4476357. For PSI-BLAST search for Goddard orthologs we searched against the NCBI nr protein database and partly PDB database. Figures that have associated raw data: 1b, 1c,

2c, 4c-e, 5c-e, Supplementary Figure 2f, 3a, 6, 7 and 8. There are no restrictions on data availability.

Field-specific reporting

Please select the one below that is the best fit for your research. If you are not sure, read the appropriate sections before making your selection.

[x] Life sciences [ ] Behavioural & social sciences [ ] Ecological, evolutionary & environmental sciences

For a reference copy of the document with all sections, see nature.com/documents/nr-reporting-summary-flat.pdf

Life sciences study design

All studies must disclose on these points even when the disclosure is negative.

Sample size	Fertility assay sample size: 30 per genotype. Testis dissection sample size: 15 per genotype. These sample sizes were chosen based on their use (and adequate power) in our prior publication on this gene
Data exclusions	No data has been excluded from the study
Replication	Fertility and quantification of cytology phenotypes are from a single replicate. Fertility deficits matched previously published results obtained through an independent method (RNAi). Qualitative antibody staining cytology was performed in two replicates. SDS-gel of Goddard expression and purification have been replicated three times. Thermal shift assay has been performed in 12 replicates.
Randomization	Males of different genotypes were paired in fertility assays with females selected from common pool.
Blinding	Researchers were not blind during data collection or analysis. The phenotypic differences would be readily apparent to any observer.

Reporting for specific materials, systems and methods

We require information from authors about some types of materials, experimental systems and methods used in many studies. Here, indicate whether each material, system or method listed is relevant to your study. If you are not sure if a list item applies to your research, read the appropriate section before selecting a response.

Materials & experimental systems

n/a	Involved in the study	
[ ]	[x]	Antibodies
[x]	[ ]	Eukaryotic cell lines
[x]	[ ]	Palaeontology and archaeology
[ ]	[x]	Animals and other organisms
[x]	[ ]	Human research participants
[x]	[ ]	Clinical data
[x]	[ ]	Dual use research of concern

Methods

n/a	Involved in the study	
[x]	[ ]	ChIP-seq
[x]	[ ]	Flow cytometry
[x]	[ ]	MRI-based neuroimaging

Antibodies

Antibodies used	Rabbit anti-HA (Cell Signaling Technology, C29F4), anti-rabbit Alexa 488 (Life Technologies, A11008)
Validation	Per the Cell Signaling Technology website, HA antibody was used to detect protein expression from functional genomic rescue construct. The antibody's specificity was validated by staining tissues from flies either carrying or not carrying the rescue construct.

Animals and other organisms

Policy information about studies involving animals; ARRIVE guidelines recommended for reporting animal research

Laboratory animals	w1118, dj-GFP./CyO(BL5417), Df(3L)ED4543 (BL8073), and Vas-Cas9(BL51323) Bloomington Drosophila Stock Center. Adult flies were used for all strains.
Wild animals	No wild animals have been used in this study
Field-collected samples	The study did not involve samples collected from field.
Ethics oversight	No ethical approval or guidance was required as no experiments has been made for which it would have been needed

Note that full information on the approval of the study protocol must also be provided in the manuscript.
